# Neutrophil-to-Lymphocyte Ratio Predicts Intravenous Immunoglobulin-Resistance in Infants Under 12-Months Old With Kawasaki Disease

**DOI:** 10.3389/fped.2019.00081

**Published:** 2019-03-19

**Authors:** Yongbing Chen, Ying Hua, Chunyu Zhang, Selena Chen, Qingyou Zhang, Ying Liao, Hui Yan, Yuli Wang, Ping Liu, Jianguang Qi, Xueqin Liu, Yonghong Chen, Chaoshu Tang, Hongfang Jin, Junbao Du

**Affiliations:** ^1^Department of Pediatrics, Peking University First Hospital, Beijing, China; ^2^Division of Biological Sciences, University of California, San Diego, San Diego, CA, United States; ^3^Department of Physiology and Pathophysiology, Health Science Center, Peking University, Beijing, China; ^4^Key Laboratory of Molecular Cardiovascular Sciences, Ministry of Education, Beijing, China

**Keywords:** Kawasaki disease, immunoglobulin, resistance, neutrophil-to-lymphocyte ratio, prediction

## Abstract

**Objective:** We evaluated the ability of peripheral blood neutrophil-to-lymphocyte ratio (NLR) to predict the intravenous immunoglobulin (IVIG) resistance in Kawasaki disease (KD) patients under 1-year of age.

**Methods:** A total of 92 KD patients under the age of 1-year and who were hospitalized in Peking University First Hospital from June 2007 to August 2016 were recruited in this study. The clinical and laboratory data were analyzed to see if peripheral blood NLR was useful for predicting the IVIG-resistance in KD.

**Results:** Totally 81 out of 92 patients were IVIG responders while 11 resistant to IVIG, with no significant difference in age, gender, ratio of the number of the incomplete to the number of complete KD, and the number of patients with coronary artery lesion between two groups (*p* > 0.05). Peripheral blood NLR was increased significantly in IVIG-resistant children compared to the IVIG responders [2.6 (interquartile range: 1.4, 3.8) vs. 1.7 (interquartile range: 0.9, 2.3), *p* = 0.039]. A cut-off value of NLR of 2.51 in KD patients younger than 1-year old yielded a sensitivity of 0.545 and specificity of 0.840, respectively, in the prediction of IVIG resistance. An area under the curve of 0.692 (95% confidence interval 0.526–0.859, *p* = 0.039) was determined.

**Conclusions:** The peripheral blood NLR ≥ 2.51 is useful to predict the IVIG resistance in KD patients younger than 1-year old.

## Introduction

Kawasaki disease (KD) is an acute systemic vasculitis first reported in 1967 with unknown etiology affecting small and medium-size arteries (1). 10–15% patients have persistence or reoccurrence of fever 36–48 h after the first dose of intravenous immunoglobulin (IVIG), which is defined as intravenous immunoglobulin resistance (2–7). In recent years, studies showed that 5–38.3% of KD patients were resistant to IVIG (3, 8–12). The prevalence of IVIG resistance was increased with greater incidence of KD (5, 9, 13, 14). Patients unresponsive to the IVIG therapy experienced a longer duration of fever or other systemic inflammatory symptoms (15). Importantly, the prevalence of coronary artery abnormalities is higher in the IVIG resistance group than in the IVIG responding group (2, 5, 8, 12, 16–21). Egami et al. and Kobayashi et al. reported a 32% incidence of coronary artery lesion (CAL) in KD cases with IVIG non-responders and only 0.8–2% with IVIG responders (9, 10). The remaining reported prevalence of CAA ranged from 17 to 43.3% for KD patients who were resistant to IVIG, while IVIG responders experienced a significantly lower incidence of CAA (2, 6, 9, 10, 13, 22, 23). Although no evidence was found to support a linear relationship between IVIG resistance and CAL, Burns et al. found an 8-fold increase in CAL prevalence in the IVIG resistant group compared to the IVIG responding group (8).

Since IVIG resistance is associated with poor outcomes in KD, especially in infants, it is important to predict IVIG resistance as early as possible to guarantee initial or rescue treatment in time. In previous studies, risk factors for IVIG non-response included male sex, age <12 months, IVIG after day 10 of illness, low hemoglobulin, thrombocytopenia, high neutrophil count, low albumin, low sodium, high C-reactive protein, impaired liver function, and high N-terminal-pro-b-type natriuretic peptide (NT-pro-BNP) (6, 9, 10, 13, 19, 20, 23–31). However, it remains necessary to search for inexpensive, easy-to-perform and useful predictors with high sensitivity and specificity for KD infants < 1-year old to predict the IVIG resistance and help the selection of suitable treatment modalities for IVIG resistant cases in clinics.

Peripheral blood neutrophil-to-lymphocyte ratio (NLR) is an indicator combining two independent inflammation markers. It serves as a useful predictor of clinical outcomes of some diseases such as surgery stress and cardiovascular diseases in recent studies (32–34). Ha et al. first reported the correlation between peripheral blood NLR and Kawasaki disease outcomes. When NLR > 5.49, the sensitivity of predicting IVIG non-response was 39% and specificity 86% (35). However, the sensitivity of prediction in Ha's paper was not very high. Cho et al. also confirmed that high NLR was associated with IVIG resistance in Kawasaki disease (36). Takeshita et al. used a combination of NLR and platelet-to-lymphocyte ratio to predict resistance to IVIG (37). In Hua's article, a new scoring system was developed to predict IVIG resistance, which included NLR as a scoring variable (38). Since there is crossover of neutrophil and lymphocyte percentage at 4–6 days and 5-years old, the range of NLR varies significantly with age, which would evidently affect its ability of prediction (39). Therefore, we attempted to determine if NLR could be used as a predictor of IVIG resistance in infants < 12 months old with Kawasaki disease.

## Subjects and Methods

### Subjects and Grouping

We retrospectively analyzed Kawasaki disease patients within 1-year old admitted to Peking University First Hospital from June 2007 to August 2016. All of the subjects fulfilled the diagnostic criteria of *Diagnosis, Treatment, and Long-Term Management of Kawasaki Disease* by AHA Scientific Statement (3), and were initially treated with IVIG (2 g/kg) plus oral aspirin. Categorization of patients into IVIG responders and IVIG resistance cases was dependent upon their persistence or reoccurrence of fever 48 h after the first dose of IVIG (5, 6).

### Methods

Basic demographic information including gender, age, and the clinical presentations were recorded. Total blood routine analysis and serum biochemistry were evaluated at a median of 5 and 6th day of fever in all patients prior to IVIG treatment, including white blood cell count, neutrophil and lymphocyte counts, platelet count, and levels of hemoglobin, C-reactive protein, liver function, serum albumin, pre-albumin, albumin-to-globulin ratio, and electrolytes. Echocardiography was performed before or after IVIG treatment, and the follow-up assessment of echocardiography was scheduled along with the course of disease. Patients experiencing persistence or reoccurrence of fever (≥38°C) 48 h after the first dose of IVIG were categorized into the IVIG resistance group after excluding other possible reasons. Otherwise, patients were grouped into the IVIG responder group (5–8). This study was approved by the Regional Ethics Committee of our hospital.

### Patient and Public Involvement

No patients were included in the design or implementation of the study. Neither were them involved in the interpretation of study results or draft of the manuscript. There are no plans to involve patients in the dissemination of results.

### Statistics

Categorical variables such as gender were analyzed by a two-sided χ^2^ test and continuous variables such as age and NLR were compared by the Student's *t*-test or Mann-Whitney *U*-test depending on whether a normal distribution was followed. For variables showing a significant difference between IVIG resistance and responder groups, cutoff values were determined by receiver operating characteristics (ROC) curves. All analyses were performed by the SPSS statistical software package ver. 18.0.

## Results

### Demographic Characteristics and Clinical Outcomes

A total of 92 patients (male 64, female 28), with 81 responding to IVIG and 11 resistant to IVIG, were enrolled in this study (Table 1). Twenty-three out of 81 IVIG responders were diagnosed as incomplete KD, while one was found in the resistant group. The proportion of incomplete KD did not differ between IVIG resistant and responder groups (*p* = 0.316). The age of patients in the IVIG resistance group was 8.2 ± 3.2 months old, while the age of patients in the IVIG responder group was 7.6 ± 3.1 months old. The main clinical manifestations of these patients consisted of fever ≥5 days, bilateral bulbar conjunctival injection, erythema, and cracking of lips and strawberry tongue. Other symptoms and signs included rash, erythema and edema of hands and feet, periungual desquamation, cervical lymphadenopathy, etc. The incidence of CAL did not differ significantly between IVIG responder and resistant groups (*p* = 0.142).

**Table 1:** Demographic and laboratory characteristics of patients.

**Groups**	**No. M/F**	**Days of fever (day)**	**Days of CBC (day)**	**Age (month)**	**WBC (×10^**9**^)**	**Neutrophil Count (×10^**9**^)**	**Lymphocyte Count (×10^**9**^)**	**NLR**	**CRP (mg/L)**	**RDW (%)**	**PLT count (×10^**9**^)**	**iKD/cKD**	**No. CAL**
IVIG resistance	10/1	11 (9, 16)	5 (4, 15)	8.2 ± 3.2	17.6 ± 7.6	10.8 ± 4.1	5.1 ± 3.4	2.6 (1.4, 3.8)	89 ± 39	13.3 (12.6, 14.7)	304 (208, 585)	1/10	6
IVIG responders	54/27	7 (6, 8)	6 (5, 7)	7.6 ± 3.1	15.3 ± 6.6	8.4 ± 5.0	5.5 ± 2.5	1.7 (0.9, 2.3)	68 ± 43	13.2 (12.6, 13.9)	412 (348, 540)	23/58	26
*p*-value	0.163	0.000	0.756	0.501	0.281	0.130	0.691	0.039	0.139	0.499	0.258	0.316	0.142

*CAL, coronary artery lesion; CBC, complete blood count; CRP, C-reactive protein; F, female; iKD/cKD, ratio of incomplete Kawasaki disease and complete Kawasaki disease, IVIG: intravenous immunoglobulin; M, male; NLR, neutrophil-to-lymphocyte ratio; No., number of patients; PLT count, platelet count; RDW, Red cell distribution width; WBC, peripheral white blood cell count*.

There was no significant difference in age and gender between the two groups (*p* = 0.501 and *p* = 0.163).

### The Changes of Peripheral Blood NLR Between the 2 Groups of KD Infants

Peripheral NLR was significantly greater in the IVIG resistance group than in the IVIG responder group [2.6 (interquartile range: 1.4, 3.8) vs. 1.7 (interquartile range: 0.9, 2.3), *p* = 0.039]. However, no statistical difference in peripheral blood white blood cell count (WBC), neutrophil count and lymphocyte count was observed between the IVIG responder and resistance group (*p* = 0.281, *p* = 0.130, and *p* = 0.691).

### Cut-Off Value of NLR Predicting IVIG Resistance

**Figure 1:**
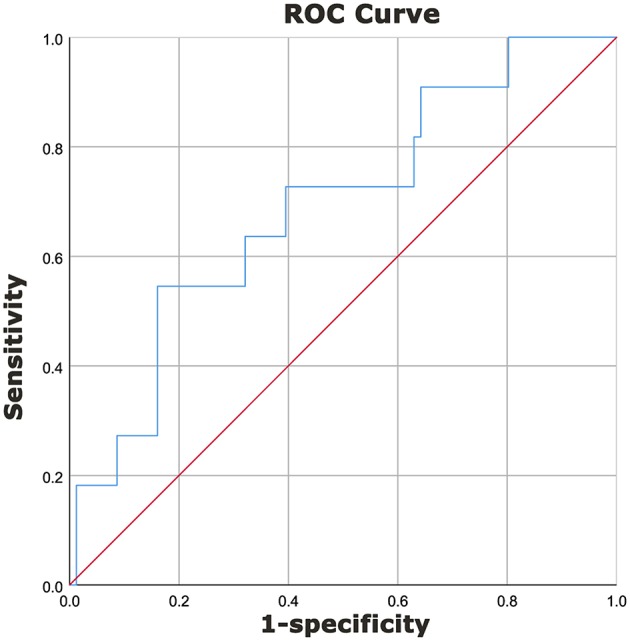
Diagnostic characteristics of NLR for prediction of IVIG resistance in KD patients <1-year of age. A receiver operating characteristics curve was performed to determine the cut-off value of NLR to predict IVIG resistance. A cut-off value of 2.51 yielded a sensitivity of 0.545 and a specificity of 0.840 for predicting IVIG resistance in patients with KD. The area under the curve (AUC) was 0.692 (95% confidence interval 0.526–0.859, *p* = 0.039).

The area under the curve (AUC) for predicting IVIG resistance with peripheral blood NLR was 0.692 (95% confidence interval 0.526–0.859, *p* = 0.039) (Figure 1). An NLR cut-off value of 2.51 yielded a sensitivity of 0.545 and specificity of 0.840 for predicting IVIG resistance in infants with KD.

## Discussions

CAL is a major complication of Kawasaki disease, which was found to be correlated with IVIG resistance. According to Miura et al. IVIG resistance was found to be significantly associated with both coronary events and major adverse cardiac events, with a hazard ratio of 2.2 (95% CI 1.4–3.6) and 3.1 (95% CI 1.5–6.3), respectively (40). This might be due to more severe inflammation-induced damage to the vascular wall in the IVIG-resistant group than in the IVIG responder group.

Around 5–38.3% of KD patients were reported resistant to IVIG in recent studies conducted (2, 5, 8, 12, 16–21). Predictors for IVIG resistance included variables in blood routine, liver function, serum electrolytes, cardiac enzymes, as well as age, gender and illness days when the initial treatment was applied (2, 5, 6, 19, 20, 23, 27, 29, 30, 35, 36, 41). There were several scoring models to predict IVIG resistance in different countries (5, 9, 10, 31). Egami scoring involved age, illness days, platelet count, ALT and CRP, yielding a sensitivity, and specificity of 78 and 75% in predicting IVIG resistance. As for Kobayashi scoring, which consisted of seven variables, the sensitivity and specificity in the prediction of resistance to IVIG were 86 and 68%, respectively. When applying Sano criteria, a sensitivity of 77% and specificity of 86% were expected in predicting the resistance to IVIG in KD (5, 9, 10, 31).

In younger age groups, the prognosis and outcome of KD have attracted great attention. As reported by Uehara and Fu et al. 19.8–32% younger patients were not responsive to IVIG (10, 12, 19). In the study by Kobayashi et al. ages <1-year old were regarded as a risk factor for IVIG resistance (9). Meanwhile, the study by Egami et al. showed that the age range within 6 months old was considered a risk factor for IVIG resistance (10). Although the clinical symptoms and signs are less typical for young patients with Kawasaki disease, reports by several groups including McCrindle et al. showed that the CAL incidence was common compared to overall data in younger-aged patients, with fewer symptoms than listed in diagnosis criteria presented by the patients when diagnosed (4, 42–48). Therefore, the early prediction of IVIG resistance is necessary in young infants with Kawasaki disease to prevent further CAL during the course. However, for younger patients, especially that of infant patients with KD who would have poorer prognosis if resistant to IVIG, no validated, and easy-to-perform biomarkers were found for IVIG resistance prediction in clinical practices (38). Therefore, it is urgent that predictive biomarkers of IVIG resistance, especially for patients <1-year old, be identified to determine a proper treatment modality for infant KD cases and therefore improve their prognosis.

The peripheral white blood cell count and its subpopulations are classic markers of inflammation. Recently however, NLR was reported to be a powerful indicator of systemic inflammation, sepsis, surgical stress and cardiovascular diseases, and cancers (32–34). Neutrophil counts reflect ongoing inflammation, while lymphocyte counts are considered a marker of immune regulatory response. NLR, a combination of neutrophils and lymphocytes, serves as a marker of balance between inflammation and immune regulation (32, 49). At the pathological level, infiltration of neutrophils in the coronary arteries is observed at the early phase, while lymphocytes dominate in the late phase. Therefore, NLR increases at the acute stage and decreases gradually during later stages of Kawasaki disease. This pattern indicates an accelerated inflammatory response in which patients resistant to IVIG might have a more severe inflammatory course.

In our study, we found that patients resistant to IVIG had a significantly higher NLR than patients <1-year of age responding to IVIG (*p* = 0.039). If NLR is no <2.51, patients with KD younger than 1-year can be predicted to be resistant to IVIG, with the sensitivity and specificity of 0.545 and 0.840, respectively. Compared to a previous study, where a predictive model involving four variables was used (38), we showed a relatively favorable specificity in the prediction of NLR which is easy-to-use and inexpensive in clinical practice.

Collectively, peripheral blood NLR ≥ 2.51 can be used as a marker to predict IVIG resistance in Kawasaki disease in patients within 1-year old. This indicator can help in predicting IVIG resistance in KD in an economical way, and it is convenient to use since blood cell count is a routine laboratory examination for both outpatients and inpatients.

However, our study had several limitations. It was of a retrospective study in nature and the sample size was small. In the future, we still need further multi-center analysis to optimize the predictor and better fit the clinical situation.

## Data Availability

The raw data supporting the conclusions of this manuscript will be made available by the authors, without undue reservation, to any qualified researcher.

## Author Contributions

YbC, YH, CT, and JD conception and design of the study. YbC, PL, YW, YH, CZ, QZ, YL, HY, JQ, XL, HJ, and JD acquisition of the data. YbC, YH, CZ, SC, QZ, YL, HY, JQ, CT, HJ, and JD analysis and interpretation of the data. YbC, YH, XL, YhC, SC, HJ, and JD drafting the manuscript. CT, HJ, JD, YbC, XL, SC, and YhC accountable for all aspects of the work, critically revising the manuscript and approval of the final manuscript. All authors had full access to all study data, read and approved the final version of the manuscript.

### Conflict of Interest Statement

The authors declare that the research was conducted in the absence of any commercial or financial relationships that could be construed as a potential conflict of interest.
